# Glycogen synthase kinase-3β is required for epithelial-mesenchymal transition and barrier dysfunction in mouse podocytes under high glucose conditions

**DOI:** 10.3892/mmr.2020.11523

**Published:** 2020-09-17

**Authors:** Jia Guo, Lili Yang, Yingjin Qiao, Zhangsuo Liu

Mol Med Rep 14: 4091-4098, 2016; DOI: 10.3892/mmr.2016.5786

Following the publication of the above article, the authors regret to report that they have encountered some difficulties in reproducing the results presented in Figs. 1 and 4 in their subsequent studies. Therefore, they have requested that the article be retracted on account of a lack of confidence in the presented data. All the named authors and the Editor of *Molecular Medicine Reports* agree to this retraction. The authors regret any inconvenience that this may cause to the readership of the Journal.

